# Leveraging Machine Learning Techniques and Engineering of Multi-Nature Features for National Daily Regional Ambulance Demand Prediction

**DOI:** 10.3390/ijerph17114179

**Published:** 2020-06-11

**Authors:** Adrian Xi Lin, Andrew Fu Wah Ho, Kang Hao Cheong, Zengxiang Li, Wentong Cai, Marcel Lucas Chee, Yih Yng Ng, Xiaokui Xiao, Marcus Eng Hock Ong

**Affiliations:** 1 School of Computer Science and Engineering, Nanyang Technological University, Singapore 639798, Singapore; linxi89@gmail.com; 2 SingHealth Duke-NUS Emergency Medicine Academic Clinical Program, Duke-National University of Singapore Medical School, Singapore 169857, Singapore; sophronesis@gmail.com; 3 SingHealth Emergency Medicine Residency Programme, Duke-National University of Singapore Medical School, Singapore 169608, Singapore; 4 Signature Research Programme in Cardiovascular & Metabolic Disorders, Duke-National University of Singapore Medical School, Singapore 169857, Singapore; 5 Science, Mathematics and Technology Cluster, Singapore University of Technology and Design (SUTD), Singapore 487372, Singapore; 6 SUTD-Massachusetts Institute of Technology International Design Centre, Singapore 487372, Singapore; 7 Institute of High Performance Computing, Agency for Science, Technology and Research, Singapore 138632, Singapore; liz@ihpc.a-star.edu.sg; 8 Lee Kong Chian School of Medicine, Nanyang Technological University, Singapore 636921, Singapore; aswtcai@ntu.edu.sg; 9 Faculty of Medicine, Nursing and Health Sciences, Monash University, VIC 3800, Australia; tche0014@student.monash.edu; 10 Emergency Medicine, Tan Tock Seng Hospital, Singapore 308433, Singapore; Yih_Yng_NG@ttsh.com.sg; 11 Home Team Medical Services Division, Ministry of Home Affairs, Singapore 179369, Singapore; 12 School of Computing, National University of Singapore, Singapore 117417, Singapore; xkxiao@nus.edu.sg; 13 Health Services & Systems Research, Duke-NUS Medical School, Singapore 169857, Singapore; marcus.ong.e.h@singhealth.com.sg; 14 Department of Emergency Medicine, Singapore General Hospital, Singapore 169608, Singapore

**Keywords:** demand prediction, ambulance deployment, emergency medical services, health informatics, emergency medicine, geospatial, complexity science, nonlinear dynamics

## Abstract

The accurate prediction of ambulance demand provides great value to emergency service providers and people living within a city. It supports the rational and dynamic allocation of ambulances and hospital staffing, and ensures patients have timely access to such resources. However, this task has been challenging due to complex multi-nature dependencies and nonlinear dynamics within ambulance demand, such as spatial characteristics involving the region of the city at which the demand is estimated, short and long-term historical demands, as well as the demographics of a region. Machine learning techniques are thus useful to quantify these characteristics of ambulance demand. However, there is generally a lack of studies that use machine learning tools for a comprehensive modeling of the important demand dependencies to predict ambulance demands. In this paper, an original and novel approach that leverages machine learning tools and extraction of features based on the multi-nature insights of ambulance demands is proposed. We experimentally evaluate the performance of next-day demand prediction across several state-of-the-art machine learning techniques and ambulance demand prediction methods, using real-world ambulatory and demographical datasets obtained from Singapore. We also provide an analysis of this ambulatory dataset and demonstrate the accuracy in modeling dependencies of different natures using various machine learning techniques.

## 1. Introduction

The accurate prediction of the daily ambulance demands across different regions of a city is of great importance to emergency service providers and its residents. Through the lens of emergency service operators, such information is valuable for a rational and dynamic deployment of ambulances of different types and increases operational effectiveness in fleet management. This in turn ensures that patients have shorter waiting times through location planning and increased ambulance availability when the need arises. This is an important goal for pre-hospital emergency medical services [1,2,3,4] and especially necessary for patients in critical conditions [5,6]. Furthermore, it helps in the efficient staffing for shifts in the hospitals, as well as early identification of any surges in ambulance demands. With the growth in focus towards data collection and analysis over the years, massive datasets of ambulance records are increasingly available for use by healthcare professionals. This encourages a stronger understanding of ambulance demands and the efficient planning of healthcare resources.

However, estimating ambulance demand through human efforts has nevertheless been challenging due to various multi-nature considerations [7]. First, ambulance demand is affected by spatial-related characteristics, such as the region at which demand is estimated. For example, a region is likely to experience a higher demand for ambulance than another region due to a larger elderly population. Moreover, a city is often demarcated by the local government into various development regions, with each having a particular purpose, e.g., financial district, or residential district. Demand is likely to be different depending on the region type. Second, ambulance demand is also affected by high-level temporal attributes, such as day of week and day of month, since the demand may often experience periodicity. Third, it is often correlated with short-term and long-term historical demands in that region. For example, if a region experiences a sudden outbreak of a disease and requires more ambulatory interventions or a sporadic mass sports event that lasts for a few days, the demand for ambulances in that region is likely to be similar over those consecutive few days. The cumulation of these multi-nature features renders ambulance demand a nonlinear dynamical system. While it may be straightforward to infer demand information from historical demands due to their temporal periodicities, it may not be as easy to do so for other features like the region identifier (ID) or day of the week. Given the inherent complexity and chaos within such systems, advanced machine learning methods will be useful for extracting insights that support the prediction of ambulance demand.

Specifically, machine learning methods have been gaining momentum over the years due to their capabilities of modelling complex patterns within data, encouraged by the advancement of computational hardware. They have demonstrated success in various areas of emergency medicine [8], such as predicting in-patient admission [9], postsurgical mortality, and intensive care unit admission [10], and in-hospital mortality of emergency department patients [11,12,13,14], all of which are complex non-linear dynamical systems. In the domain of ambulance-related research, machine learning has been considered for ambulance travel time estimation [15,16], location selection for ambulance stations [17], and demand prediction [17,18]. Despite the progress that has had been made, there is generally a lack of studies that consider such methods for the ambulance demand prediction problem.

Furthermore, existing work may consider machine learning methods for ambulance demand prediction, they generally do not incorporate in a sufficiently comprehensive manner the various types of dependencies affecting ambulance demand. They either consider prediction of the whole city [19] or predict demand at equally sized square grids [7,20], which is not reflective of the actual regionalization by the local government. In other instances, the focus is only on prediction of demands at some, but not all, regions of a city [18]. In this paper, we propose an original and novel approach that leverages a massive dataset of historical ambulance demand records to model the multi-nature dependencies of ambulance demand for predicting the next-day demand at all regions of a city-state. Our approach elicits useful insights that represent each of the different types of dependencies. Then, it utilizes a machine learning model to learn these dependencies for prediction. We evaluate the performance across several state-of-the-art machine learning techniques, using real-world ambulance demand datasets recorded by Singapore Civil Defence Force (SCDF).

## 2. Materials and Methods

### 2.1. Data Sources

In this study, we make use of a dataset obtained from the SCDF that includes all the ambulance calls in the city-state, Singapore, from 2006 to 2016. SCDF is the single national emergency ambulance provider which manages a fleet of around 60 ambulances in 2016 [21]. SCDF activates these ambulances by a centralized “995” dispatching system and does not charge for any emergency cases it conveys to hospitals [22]. Each ambulance call in the dataset corresponds to an incident, which has the following characteristics: time of incident, ambulance origin station, incident classification, incident subclass, patient incident subclass, patient’s emergency status, patient’s year of birth, ambulance destination hospital, patient location common name, patient location postal code, patient location street, incident location latitude, incident location longitude, and gender. To obtain the regions and map of Singapore, we make use of the 2010 Planning Area Census by the Singapore government, which consists of every Development Guide Plan (DGP) region (similar conceptually to census tracts). We also leverage datasets obtained from polyclinics in Singapore to extract useful demographical information such as the population count of people above a certain age at each region of the city-state. Finally, we consider additional socioeconomic information from The Census of Population conducted by the Singapore Department of Statistics in 2010, which is the most recent one available. Such census is conducted once every ten years and is based on a person’s place of usual residence.

### 2.2. Model Overview

**Figure 1:**
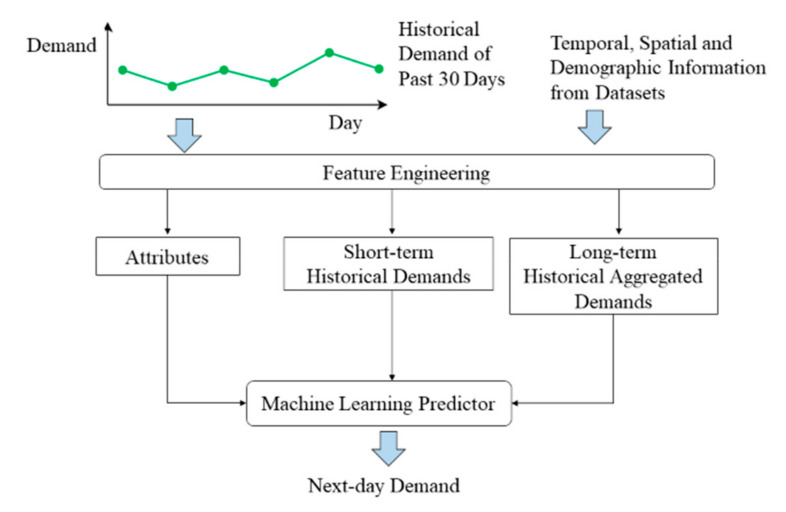
Approach overview schematic.

Using the above-mentioned datasets, we design an approach that involves a Feature Engineering stage and a prediction stage using a Machine Learning Predictor. Figure 1 shows an overview schematic of the approach. In the following sections, we elaborate each of these stages in details.

### 2.3. Feature Engineering

Data processing is first carried out on the SCDF dataset to generate the aggregated demand, i.e., number of calls, and several associated features of each region of each day from 2006 to 2016. We denote the engineered dataset as SCDF-Engineered. Each data sample in SCDF-Engineered consists of the aggregated ambulance demand at a particular region of a day within 2006 to 2016, which is the outcome of interest. It also includes several features associated with that region on the particular day. Specifically, these features fall under three classes: Attributes, Short-term Historical Demands, and Long-term Historical Aggregated Demands.

The description of the features in each of the three classes are as follows:

- Attributes. These are categorical features that provide high-level information about the record. These features are multi-nature and can be further classified into (1) spatial, (2) temporal, and (3) demographic attributes. Specifically, the spatial attributes consist of the region ID, which is a number that uniquely identifies each DGP region. The rationale behind its inclusion is to differentiate the regions within Singapore, since different regions may have different demand characteristics. For example, a region with more elderly people may experience a higher demand than another region with mostly young people. The temporal attributes consist of the following features: day of week, day of month, and month of year. These are included to account for the periodicity of ambulance demands. Finally, since the demand at a region may be higher if it has more people who are older in age, we also consider a demographic attribute: the total no. of people in that region who are aged 50 and above on that particular year
- Short-term Historical Demands. These features are demands at a region over each of the previous 7 days. These 7 continuous features are considered to account for the correlations between the demands of a particular day with that of the previous days. For example, a sudden spike in the dengue mosquitos’ population at a region may result in the rise of dengue-related cases over a few consecutive days.
- Long-term Historical Aggregated Demands. These features consist of the total demand at a region over the past 30 days, the total demand over the past 7 days, the total demand of the week up until the sample date, and the total demand of the month up until the sample date. These aggregated demands are included to account for the demand on the broader scale without the higher variances present in short-term demands. For example, a region may experience a high short-term historical demand solely due to a recent occurrence of a large-scale traffic accident but does not typically have high demands as it is not a populous area.

Apart from SCDF-Engineered, we also further build a dataset SCDF-Engineered-Socio. The rationale behind building this dataset is to explore whether ambulance demand has any correlations with the socio-economic characteristics of the people in a region. Similar to SCDF-Engineered, each data sample in SCDF-Engineered-Socio contains all the features in Attributes, Short-term Historical Demands, and Long-term Aggregated Demands. However, additional socioeconomic features of each region obtained from The Census of Population is also included in this dataset. Specifically, SCDF-Engineered-Socio also considers the following additional features: number of residents who travel by buses, number of residents who travel by cars, number of residents who travel by taxis, number of residents who travel by trains, number of residents who are in active employment, number of residents who are unemployed, number of residents who are tenants, and number of residents who are home owners. Since the socioeconomic information is only available for a subset of regions in Singapore, SCDF-Engineered-Socio contains only data samples from these regions.

As observed, the features considered so far are in their entirety, multi-nature. However, each feature represents a piece of information of only a single nature and does not consider the impacts of mixed features. For example, the feature day of week only reveals temporal information about a record, but it does not reveal its relationship with the region at which the record corresponds to. In order to study the impact of mixed features, we further engineer composite features based on the existing features generated. Specifically, we consider spatiotemporal features, and create the following composite features: unique ID that represents (region ID, day of week, day of month, month of year), unique ID that represents (region ID, day of week), unique ID representing (region ID, day of month), and unique ID that represents (region ID, month of year). For evaluation purpose of such composite features, we create a separate dataset SCDF-Engineered-Spatiotemporal (SCDF-Engineered-ST). This dataset includes the same features present in SCDF-Engineered, as well as the engineered spatiotemporal composite features.

### 2.4. Key Implementation Details of Feature Engineering

The key component of our feature engineering lies in the extracting of the short and long-term demands, since other features can be obtained either directly from the raw dataset in the case of features like the day of week, or mapped easily using a third-party Application Programming Interface (API) in the case of other features like region ID. To extract these demand features, we first use simple aggregations to transform the SCDF dataset into a dataset that records the total daily demand of each region and sort these demands in a chronological order.

**Figure 2:**
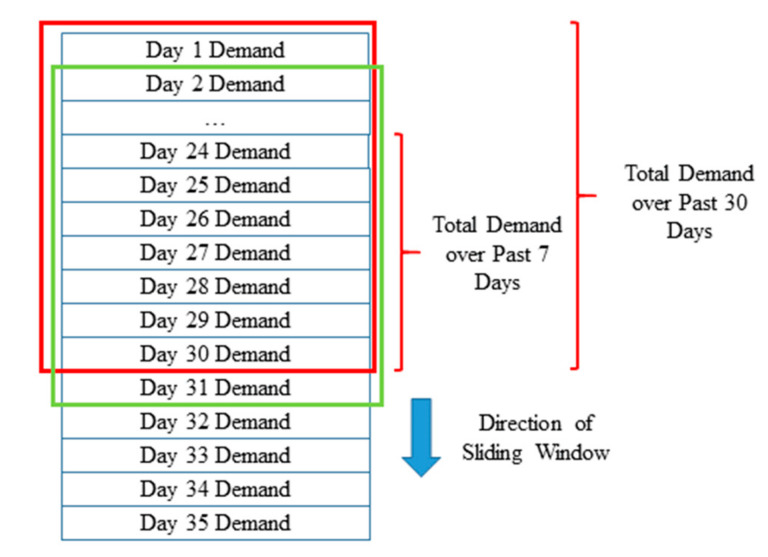
Sliding window for extraction of demand features.

Then, we use a sliding-window-based approach to obtain the relevant demand variations for each data sample. Figure 2 demonstrates an example of such a process. The red box represents a sliding window that essentially contains the historical ambulance demand values over each of the past 30 days. Within this window, the relevant short-term historical demand features are extracted. The green box represents a sliding window that considers the historical demand over each of the past 7 days. Within this window the long-term historical and aggregated demand features are extracted. As mentioned, the time frames chosen are 7 and 30 days to account for the weekly and monthly demand periodicities respectively. Once this is completed, the two sliding windows move on to the next time step to extract the similar features for the next day. This process is then conducted for all regions of the city to get all the data samples used in this study.

### 2.5. Primary Outcome

The outcome of interest is the next-day aggregated ambulance demand at a DGP region of Singapore. This demand may arise from incidents of different emergency statuses, i.e., Dead on Arrival, Emergency Critical, Emergency (Non-ambulatory), Emergency (Ambulatory), and Non-emergency. It is also agnostic to trauma and medical incidents and incidents where assistance were not required. Hence, it is a regression task from the machine learning point of view.

### 2.6. Machine Learning Methods Considered

Given the engineered dataset, we want to train a machine learning model that predicts the demand by leveraging the above-mentioned features. To this end, we make use of the hold-out evaluation technique. Specifically, the data samples in SCDF-Engineered from 2006–2015 are used for model training, while the samples from 2016 are used for model validating. The similar separation is done for SCDF-Engineered-Socio. Several methods are considered, and they are chosen because they are either typically effective for regression problems or had been previously considered in existing work. The methods are as follows:

- Regional Moving Average. This method estimates the next-day demand at a region simply by taking the average of the daily demand values over the past 7 days at this region.
- Linear Regression. This method is a popular regression method that finds the best-fit hyperplane across the multi-feature data samples [23]. It assumes a linear relationship between the dependent variable, i.e., demand, and the independent variables, i.e., features. To model this relationship, the mean square error function is first considered as a loss function. Then, the gradient descent algorithm is used to iteratively find the minimum of this function and also the resulting hyperplane. The coefficients of this hyperplane represent the degree of impact each feature has on the predicted value. To accurately represent the categorical features, i.e., Attributes, one-hot encoding is used during preprocessing. Min-max scaling is also applied on the continuous features. This method is applied using the Python Scikit-Learn library [24].
- Support Vector Regression (SVR). SVR is a support-vector machine that performs regression by finding a hyperplane, i.e., support vector, that fits as many points as possible within a space that is bounded by two boundary hyperplanes parallel to this support vector. Unlike Linear Regression, SVR typically finds the best-fitting hyperplane in the higher dimensions. To this end, it utilizes a kernel, which is a function that maps lower-dimensional data points to higher-dimension data points. The advantage of doing so is that it allows the method to capture certain non-linear relationships, which may not be possible with Linear Regression. SVR has been demonstrated to be one of the more effective machine learning approaches for predicting ambulance demand in [18]. Similar to Linear Regression, we apply this method using the Python Scikit-Learn library [24] and process the categorical features with one-hot encoding.
- Multi-layer Perceptron (MLP). This method is an artificial neural architecture that has been explored and demonstrated in [19] to be an improvement over the traditional ambulance demand prediction method. The MLP is a standard neural architecture that is essentially made up of a sequence of linear layers. In this baseline, the size of the hidden layer is equal to that of the input layer, and 3 hidden layers are considered in total. Furthermore, the loss function used for the training of the model is the squared loss function. The learning rate used is 0.01, and the activation function used is the ReLU function. This method is also applied from the Python Scikit-Learn library [24].
- Radial Basis Function Network (RBFN). We also consider the Radial Basis Function (RBF) network, a variant of the artificial neural network (ANN), for comparison. Unlike a typical MLP network, a RBFN consists of three layers: an input layer, a linear output layer, and a hidden layer that uses the non-linear radial basis function as the activation function. It has been demonstrated to be more effective than traditional MLPs in certain problems [25].
- Light Gradient Boosting Machine (LightGBM). LightGBM [26] is one of the most efficient and high-performing gradient-boosting decision tree methods. The key idea behind such gradient-boosting methods is that they consider the ensemble of various individual regression trees to fine-tune the accuracy of prediction. This is achieved by sequentially combining the trees such that each tree fits to the residual of the previous tree it is extended from. The input for this method is similar to that of the previous methods, with the exception that attributes are specified as categorical features in the program. Furthermore, the specific key settings considered in this work are as follows. (1) Number of trees, 2000; (2) number of leaves, 31; (3) learning rate, 0.005; (4) feature fraction, 0.8. The boosting approach considered is gradient boosting decision tree. This method can be applied by using the LightGBM library [26] in Python.

The error metrics used in the experiments are weighted absolute percentage error (WAPE), mean absolute error (MAE), and mean squared error (MSE) [27,28]. WAPE is used as an error metric instead of the mean absolute percentage error (MAPE). This is because the ground-truth demand at a region may sometimes be zero, which results in the zero-division error if MAPE is used. Specifically, the formulation for WAPE is as follows:

(1) $$WAPE=(\frac{\sum |A-F|}{\sum A}),$$

where *A* denotes a ground-truth demand, and *F* denotes its corresponding predicted value.

In our implementation, feature engineering is carried out using Python (version 2.7.16, Python Software Foundation, Delaware, USA). To map an incident to its corresponding region, the Shapely library is used [29]. The above-mentioned data-mining regression methods are built using Python Scikit-Learn library (version 0.20.0) [24], and LightGBM library (version 2.2.3) [26]. We also make use of QGIS (Open Source Geospatial Foundation, Beaverton, Oregon, USA) for spatial-related visualizations.

## 3. Results

Table 1 shows the key characteristics of the SCDF demand dataset. Specifically, it shows different compositions of the dataset, over the following category types: incident year, incident classification, incident subclass, patient incident subclass, patient’s birth year, and patient’s gender. As observed, there is a general increasing trend for ambulance demands from 2006 to 2016. The median age (based on the age of the patient by the year-end of the incident) is 55 and largely between 34 and 73. This reveals that more than half of the incidents occurred to middle-aged and elderly people and that most of the incidents happened to people who were at least young adults.

On the biennial level, the patient ages generally increase from 2006 to 2016. In terms of incident classification, the majority of the incidents were trauma in nature. The analysis of patient incident subclass shows that the majority of calls were due to problems associated with the nervous system. However, there is also a large proportion of calls where the patient was uninjured or did not have any medical complaints. Other major sources of calls were problems associated with the bone/connective tissue, respiratory system, and cardiovascular system. This is in line with the idea that the increasing demand may be due to an increasingly aging population since problems at these parts of the body tend to be associated with the elderly.

Preprocessing and feature engineering are conducted on the SCDF dataset to build SCDF-Engineered. Table 2 shows some of the characteristics of this engineered dataset. The overall mean daily regional demand is 6.33 and ranges largely from 0 to 10. The mean of the total past-7-days regional demand is 44 and largely ranges from 4 to 69. This is in contrast to the total past-30-days demand, where the mean, the first quartile, and the last quartile are 190, 20, and 294, respectively. Within SCDF-Engineered, each record consists of the Attributes, Short-term Historical Demands, and Long-term Historical Aggregated Demands features, as per the descriptions in Section 2. Since each record in SCDF-Engineered is specific to a day and a region; the ground-truth values associated with this record is simply the demand on that day and in that region. These ground-truth values are used as the target variables during model training (resp. validation), using records from 2006–2015 (resp. 2016) in SCDF-Engineered.

**Figure 3:**
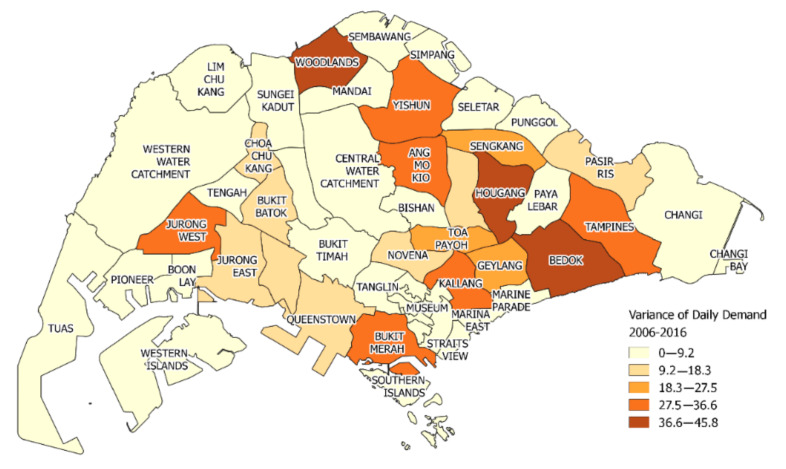
Map of regional variance of daily demand in Singapore from 2006 to 2016.

Figure 3 shows the variance of the daily demands of each region over the days of 2006–2016. As observed, the demand variances vary across the regions. This highlights differences in demand behaviors across different regions and the importance of considering region ID as a feature to account for such differences.

Table 3 shows the accuracies of the five methods compared on SCDF-Engineered, with the best results highlighted in bold. As observed, the performance of both Linear Regression and LightGBM are the best and comparable with each other, with the former having a slight edge in terms of the MSE metric. The regional moving average is the worst performing method, while the performances of SVR, MLP, and RBFN are somewhere in the middle of all methods compared. Comparing MLP and RBFN, the former also demonstrates a stronger performance for the problem we are solving. Although Linear Regression is one of the best-performing methods, it may be subjected to overfitting, since according to analysis, the mean coefficient value is 3.9 × 1011, and the interquartile range is between −1.55 and 2.7 × 1011. As such, Linear Regression may not be a suitable model due to the instability introduced through the largely varying coefficients that arise from overfitting. This implies that it may not perform as well on other datasets. Since Gradient-boosting Decision Tree is also highly effective for structured data, e.g., table of features as in our case, such methods are preferred in our context. Due to its effectiveness, LightGBM is specifically chosen.

Table 4 shows the gain-based importance of the features derived from the training process of LightGBM, as well as the mean absolute SHapely Additive exPlanations (SHAP) value of each feature. The SHAP value essentially assigns each feature an importance value for each prediction [30]. To obtain the overall importance for each feature, the mean absolute SHAP value is considered, where a larger value represents a greater feature importance. In terms of the relative importance of a feature among all considered features, both the LightGBM’s gain-based importance and mean absolute SHAP value are observed to be in agreement with each other.

The most important features are the total demand over the past 30 days and the total demand over the past 7 days in that region. This highlights the importance of considering long-term historical aggregated demands. What follows is the ID of the region at which the demand is predicted. This demonstrates the importance of differentiating a region from other regions, since they may have vastly different demand characteristics, as shown in Figure 2. The total number of people aged 50 and above in the region of the particular year is also considered important. This is in line with our intuition that people who are older in age are more likely to require emergency assistance than younger ones. The day of month, day of week, and month of year are also significant features, since there are periodicities within the ambulance demands. Finally, the demand at the region on each of the past 7 days contributes to the estimation by a fair extent.

To demonstrate the effects of regional socioeconomic data, we evaluate how the best-performing model of Table 3 performs when these regional socioeconomic features are included. Table 5 compares the accuracy of the prediction when LightGBM is applied on SCDF-Engineered-Socio, with the accuracy when these socioeconomic features are excluded from SCDF-Engineered-Socio. However, as observed, including additional socioeconomic features does not improve the prediction. A reason may be because these features are constant throughout all the years within the dataset. Any insights that these regional socioeconomic features provide may have already been represented by the region ID, which is one of the most important features according to Table 3. This is unlike the regional demographic feature present in the Attributes, where the total number of people aged 50 is different every year.

To evaluate the impact of adding spatiotemporal composite features, we also apply LightGBM on SCDF-Engineered-ST. The resulting accuracies are WAPE = 24.7%, MAE = 2.11, and MSE = 10.4. As observed, these accuracies are worse than when no composite features are used. The reason may be because even though the features consider the spatiotemporal characteristics of a record, they may be noise to LightGBM, which algorithmically considers the mixed effects of different features in a finer-grained manner, by merits of the algorithm. This further highlights the benefits of using gradient-boosting machines for such problems.

## 4. Discussion

This study analyses a large city-scale ambulance demand dataset using machine learning algorithms to further develop a daily regional demand prediction tool. Our work is novel because it is the first reported study in Singapore to leverage machine learning in the development of tools that assist in the planning of emergency response resources. To this end, it considers various multi-nature dependencies of ambulance demands. This motivates future work in conducting machine learning-based analysis on datasets of similar types.

Our solution considers the engineering of various attributional features, short-term historical demands, and long-term historical aggregated demands. LightGBM is then applied on these features for the prediction of demand. Other methods either do not perform as well, or encounter problems like overfitting, as in the case of Linear Regression. As such, LightGBM remains the top choice in our solution. The reason why an ensemble model like LightGBM performs better than individual ones like linear regression may be that it combines various independent models via the gradient boosting approach. Specifically, each model within LightGBM is a regression tree, which in itself is more suitable than models like Linear Regression in capturing the non-linear dependencies of ambulance demand. The key idea behind gradient boosting is that prediction can be refined by adding these trees one at a time while using a gradient descent procedure.

The proposed features contribute in varying degrees to the model training in LightGBM. The most important features are the ID of the region at which demand is predicted, long-term historical aggregated demand features, day of month, and number of people aged 50 and above. With the results obtained from this study, it provides emergency healthcare resource planners additional insights on how different features affect the demand at a region for effective ambulatory resource planning in the future. For example, understanding that the region of the city-state is one of the greatest determinants of demands allows the planners to dispatch ambulances in a finer-grained manner. Furthermore, understanding that the demand at a region also strongly depends on its long-term historical demand and number of people aged above 50 encourages planners to focus suitable amount of resources to regions based on the historical incidents that occur at the region. It also encourages paying more attention to the demographical changes in each region.

While the accuracy of around 25% is considered satisfactory, it may not be as high when compared to the prediction of other vehicles like taxis/on-demand vehicles [31,32]. We note that the reason for this may be that the regional demand for vehicles like taxis is typically much larger than that of ambulances to begin with, which in our case is only around 6 per day per region. As such, the prediction percentage error in our case is more likely to be larger, due to the relatively much smaller size of the target outcome used in the machine learning training and prediction. The periodicity of ambulance demand is not as strong as other types of vehicles like taxis. While the demands for taxis or private hires may be highly dependent on the days of the week, similar results cannot be inferred for ambulances. This motivates us to consider various external data sources in our future work, e.g., weather conditions, to model the other possible dependencies that may affect the ambulance demand. Furthermore, our solution is a preliminary take on this problem in Singapore, and it predicts the demands only under certain typical conditions. Although there are peaks and troughs in demands every now and then, these values are in no way near the extremes that happen during very large-scale incidents, e.g., epidemics, haze [33,34,35], and diurnal temperature changes. A potential area of improvement is to make use of historical data to model the demand at such extreme cases of large-scale incidents.

We have seen applications of artificial intelligence and machine learning techniques across different disciplines [36,37,38,39]. Our work here focuses on health services research, which has not gained much attention until now. Other than predicting the daily demand, future work involves further optimization to investigate finer-grained demands, e.g., hourly. However, as we look at increasing the granularity of analysis to identify “micro-trends” and pockets of demand, which may potentially be matched with better optimized placements or additional ambulance staffing, the operational limitations of block scheduling need to be considered. It may not be practical to call up a person to work for just 1–2 h instead of the typical 8 to 12 h shifts. The emergency medical services (EMS) systems may also have more rigid shift patterns, and this may limit the flexibility for optimization. Furthermore, given that the mean daily regional demand is low, considering a finer-grained timescale may result in the issue of data sparsity that inhibits accurate model trainings. These considerations are beyond the scope of the current study and will form part of our future investigation.

This study demonstrates the usage of a single-source vehicle, i.e., ambulance, dataset for building of a solution that models and predicts the ambulance demand in the regions of a city-state. For future work, we may additionally consider the insights obtainable from other vehicle datasets, e.g., taxi trajectories or public transportation smart card data. For example, a potential direction is to further consider the accessibility of each region to its respective nearest hospitals or clinics using certain metrics, e.g., average travel distance/duration of trips originating from a region and ending at a hospital. The idea is that if accessibility by other forms of transportation is higher, it gives people more alternatives for traveling to the hospitals instead of focusing solely on ambulance, especially for non-critical incidents. This may in turn affect the demand of ambulances in that particular region. Furthermore, leveraging geospatial datasets from other vehicles also allows us to understand the medical demand of people within a region. If a particular region sees on average a larger number of people traveling to the hospitals/clinics via public transportation or taxis than another region, an assumption can possibly be made that the former region tends to house more people who may require medical care than the latter. While this does not necessarily imply a higher ambulance demand, which focuses on more urgent cases, a potential exploration on the correlations between these two pieces of information can also be considered for future work.

## 5. Conclusions

In this study, we have utilized a 10-year city-wide emergency ambulance dataset to predict ambulance demand. The forecasting capability presented here is important because it enables informed resource and ambulance demand and is applicable across hospitals and general medical facilities. Several machine learning techniques are compared: Regional Moving Average, Linear Regression, Support Vector Regression, Multi-layer Perceptron, and LightGBM. Based on the preliminary work carried out here, LightGBM is found to perform the best. The most important features are the total demand over the past 30 days and the total demand over the past 7 days in that region.

## Figures and Tables

**Table 1:** Characteristics of ambulance demand dataset.

Characteristics	Value
Incident Year	
2006–2007	190,608 (13.6%)
2008–2009	216,841 (15.5%)
2010–2011	237,451 (17.0%)
2012–2013	268,596 (19.2%)
2014–2016	311,251 (22.3%)
2016	172,009 (12.3%)
Patient Age (yrs)	55 (34–73)
2006–2007	51 (32–71)
2008–2009	52 (32–71)
2010–2011	53 (33–72)
2012–2013	56 (35–73)
2014–2016	57 (36–74)
2016	58 (36–75)
Incident Classification	
Medical	968,375 (69.3%)
Trauma	391,986 (28.1%)
Assistance Not Required	35,460 (2.54%)
Patient Incident Subclass	
Nervous System	381,634 (27.3%)
No Medical Complaint/Un-Injured	385,430 (27.6%)
Bone/Connective Tissue	116,173 (8.32%)
Alcoholic Intoxication	25,865 (1.85%)
Respiratory System	132.163 (9.46%)
Reproductive System	117,21 (0.839%)
Cardiovascular System	115,587 (8.28%)
Digestive System	98,129 (7.03%)
Poisoning/Drug Overdose	6791 (0.486%)
Ear/Nose/Throat/Eye Condition	5601 (0.401%)
Kidney/Urinary System	16,433 (1.18%)
Blood Related	5590 (0.400%)
Maternity/Childbirth	5062 (0.362 %)
Liver/Biliary Tract	1438 (0.103%)
Psychiatric Emergencies	4413 (0.316%)
Endocrine System	31,850 (2.28%)
Infectious Disease/Disorder of Skin	4649 (0.333%)
Others	35,240 (2.52%)
Unknown	9474 (0.678%)
Unclassified	2705 (0.194%)
Gender	
Male	838,737 (60.0%)
Female	554,237 (39.7%)
Unclassified	2163 (0.213%)

For continuous variables, data is presented in medians and interquartile ranges. For categorical variables, data is presented in frequencies and percentages.

**Table 2:** Characteristics of engineered dataset.

Characteristics	Value
Daily Regional Demand	6.33 (0–10)
Total Regional Demands over Past 7 Days	44. (4–69)
Total Regional Demands over Past 30 Days	190 (20–294)

Data is presented in means and interquartile ranges.

**Table 3:** Method accuracy comparisons

Method	WAPE (%)	MAE	MSE
Regional Moving Average	25.8	2.20	11.2
Linear Regression	**24.5**	**2.09**	**10.1**
MLP	24.6	2.10	**10.1**
RBFN	25.1	2.14	10.8
SVR	25.2	2.15	11.2
LightGBM	24.5	2.09	10.2

Bold indicates the best results for each column. WAPE: weighted absolute percentage error; MAE: mean absolute error; MSE: mean squared error; MLP: multilayer perceptron; RBFN: Radial Basis Function network; SVR: Support Vector Regression; LightGBM: Light Gradient Boosting Machine.

**Table 4:** Feature importance.

Feature	Gain-Based Importance	Mean Absolute SHAP Value
Region ID	14,121,071	0.230
Day of Week	844,283	0.069
Day of Month	2,400,462	0.043
Month of Year	723,054	0.031
Demand 1 Day Ago	308,590	0.022
Demand 2 Days Ago	138,543	0.011
Demand 3 Days Ago	159,649	0.012
Demand 4 Days Ago	209,771	0.014
Demand 5 Days Ago	144,138	0.015
Demand 6 Days Ago	146,966	0.009
Demand 7 Days Ago	432,136	0.022
Total Demand of the Week up to the Data Sample Day	1,368,848	0.101
Total Demand of the Month up to the Data Sample Day	82,847	0.005
Total Demand over Past 30 Days	820,758,893	4.626
Total Demand over Past 7 Days	77,034,386	0.466
Total Number of People Aged 50 and Above in the Year	2,528,021	0.223

SHAP: SHapley Additive exPlanations; ID: identifier.

**Table 5:** Accuracy comparisons on inclusion/exclusion of regional socioeconomic features.

Dataset	WAPE (%)	MAE	MSE
SCDF-Engineered-Socio	22.0	3.00	16.3
SCDF-Engineered-Socio, excluding regional socioeconomic features	22.0	3.00	16.3

WAPE: weighted absolute percentage error; MAE: mean absolute error; MSE: mean squared error; SCDF: Singapore Civil Defence Force.
